# Hepatotoxicity of a Cannabidiol-Rich Cannabis Extract in the Mouse Model

**DOI:** 10.3390/molecules24091694

**Published:** 2019-04-30

**Authors:** Laura E. Ewing, Charles M. Skinner, Charles M. Quick, Stefanie Kennon-McGill, Mitchell R. McGill, Larry A. Walker, Mahmoud A. ElSohly, Bill J. Gurley, Igor Koturbash

**Affiliations:** 1 Department of Environmental and Occupational Health, University of Arkansas for Medical Sciences, Little Rock, AR 72205, USA; leewing@uams.edu (L.E.E.); cmskinner@uams.edu (C.M.S.); skennonmcgill@uams.edu (S.K.-M.); mmcgill@uams.edu (M.R.M.); 2 Department of Pharmacology and Toxicology, University of Arkansas for Medical Sciences, Little Rock, AR 72205, USA; 3 Center for Dietary Supplements Research, University of Arkansas for Medical Sciences, Little Rock, AR 72205, USA; gurleybillyj@uams.edu; 4 Department of Pathology, University of Arkansas for Medical Sciences, Little Rock, AR 72205, USA; quickcharlesm@uams.edu; 5 National Center for Natural Products Research, University of Mississippi, University, MS 38677, USA; lwalker@olemiss.edu (L.A.W.); melsohly@olemiss.edu (M.A.E.); 6 ElSohly Laboratories, Inc. (ELI), Oxford, MS 38655, USA; 7 Department of Pharmaceutics and Drug Delivery, School of Pharmacy, University of Mississippi, University, MS 38677, USA; 8 Department of Pharmaceutical Sciences, University of Arkansas for Medical Sciences, Little Rock, AR 72223, USA

**Keywords:** cannabidiol, hepatotoxicity, liver injury, natural products, phytochemical

## Abstract

The goal of this study was to investigate Cannabidiol (CBD) hepatotoxicity in 8-week-old male B6C3F_1_ mice. Animals were gavaged with either 0, 246, 738, or 2460 mg/kg of CBD (acute toxicity, 24 h) or with daily doses of 0, 61.5, 184.5, or 615 mg/kg for 10 days (sub-acute toxicity). These doses were the allometrically scaled mouse equivalent doses (MED) of the maximum recommended human maintenance dose of CBD in EPIDIOLEX^®^ (20 mg/kg). In the acute study, significant increases in liver-to-body weight (LBW) ratios, plasma ALT, AST, and total bilirubin were observed for the 2460 mg/kg dose. In the sub-acute study, 75% of mice gavaged with 615 mg/kg developed a moribund condition between days three and four. As in the acute phase, 615 mg/kg CBD increased LBW ratios, ALT, AST, and total bilirubin. Hepatotoxicity gene expression arrays revealed that CBD differentially regulated more than 50 genes, many of which were linked to oxidative stress responses, lipid metabolism pathways and drug metabolizing enzymes. In conclusion, CBD exhibited clear signs of hepatotoxicity, possibly of a cholestatic nature. The involvement of numerous pathways associated with lipid and xenobiotic metabolism raises serious concerns about potential drug interactions as well as the safety of CBD.

## 1. Introduction

Cannabidiol (CBD) is a non-psychotropic phytochemical present in *Cannabis sativa* that has gained significant popularity over the last decade. It is a major component of EPIDIOLEX^®^, a drug indicated for the treatment of drug-resistant epileptic seizures associated with Dravet and Lennox-Gastaut syndromes [1,2]. CBD has also been proposed as treatment for a number of other neuropsychiatric disorders for which clinical trials are currently ongoing [3].

CBD has also been marketed for a wide range of other indications, including ‘anti-cancer’, ‘anti-inflammatory’, ‘sleep promotion’, ‘relaxation’, ‘normal cartilage and joint function’, ‘antioxidant effects’, and ‘pain management’ just to name a few. The vast majority of those effects, however, were documented either in vitro or in clinical trials with equivocal results [4,5]. Apart from its purported salutary effects, accumulating evidence from pre-clinical in vivo studies and large-scale clinical trials, implies that CBD may elicit several potentially negative health outcomes. Specifically, numerous reports have demonstrated neurological, cardiovascular and reproductive toxicities subsequent to CBD use [6,7,8,9,10,11,12,13,14]. The authors of a large clinical trial that utilized CBD (dose regimen 2.5–30 mg/kg/day) to treat 278 patients with Dravet syndrome reported adverse events in 93% of subjects [15]. Another recent study inferred a strong genotoxic potential for CBD at concentrations commonly detected in human blood [16]. Furthermore, CBD may have a high drug interaction potential as it modulates numerous cytochrome P450 enzymes responsible for xenobiotic metabolism [17,18,19,20,21].

Of particular concern is the risk for CBD-induced hepatotoxicity [22]. Animal studies have reported increased liver weights in rhesus monkeys and elevated liver enzymes in dogs when CBD was administered at doses as low as 2 mg/kg of body weight [14,23]. In recent clinical trials, elevated liver enzymes were observed in 5–20% of patients treated with CBD, and a few patients were withdrawn due to the threat of fulminant liver failure [1,2,24]. 

The number of ‘CBD-containing’ products, available mostly online, is growing exponentially. However, the U. S. Food and Drug Administration (FDA) prohibits sales of CBD as a dietary supplement or food ingredient on the grounds that any ‘article’ that has been approved as a new drug or authorized for investigation as a new drug cannot be marketed as an ingredient in dietary supplements or conventional foods per the Food, Drug, & Cosmetic Act (FDCA) [21 U.S.C. §321(ff)(3)(B) and 21 U.S.C. §331(II), respectively] [25]. Furthermore, a clear regulatory oversight exists which has led to an uncontrolled CBD market that, in turn, threatens the health of a trusting general public. For instance, in a series of tests performed by the FDA on a panel of ‘CBD-containing products’, a large fraction either did not contain the label-claimed quantity of CBD or they were contaminated with Δ9-tetrahydrocannabidiol (THC) [26]. Furthermore, a recent independent analysis performed by CosumerLab.com, revealed that CBD doses in commercially-available products ranged from as little as 2.2 mg to as much as 22.3 mg, further amplifying concerns of potential toxicity [27]. 

As expansion of the CBD market seems inevitable, additional scientific studies are needed in order to support any required regulatory actions. For instance, if CBD is to be considered as a food additive, it will have to be filed as a new dietary ingredient (NDI) or a GRAS (generally recognized as safe) notice will need to be submitted to FDA. The latter will require a number of toxicity studies, the majority of which, in the case of CBD, remain to be performed. Analysis of genotoxic potential of CBD, the first toxicity test recommended by the FDA, was recently performed and the results published [16]. Therefore, we proceeded to the next set of recommended tests designed to address the short-term toxicity of a CBD-rich extract in a rodent model. Since liver injury is the primary concern for CBD, this study was designed to investigate the hepatotoxicity potential of CBD. The data collected in this study will provide important information for both industry and regulatory agencies in regards to the short-term toxicity of CBD. Furthermore, the results of these studies will aid in selecting appropriate models and doses for long-term studies (i.e., sub-chronic and chronic toxicity as well as carcinogenicity and reproductive toxicity studies) as well as the determination of a no observable effect level (NOEL) for selected endpoints.

## 2. Results

### 2.1. Acute Toxicity Study

For acute toxicity studies, a dose of 246 mg/kg was chosen as an initial dose as this is a MED analogous to those used in recent clinical trials (MED of 20 mg/kg CBD) [1,2]. Subsequently, we used doses of 738 and 2460 mg/kg CBD as 3× and 10× doses, respectively. 

**Figure 1:**
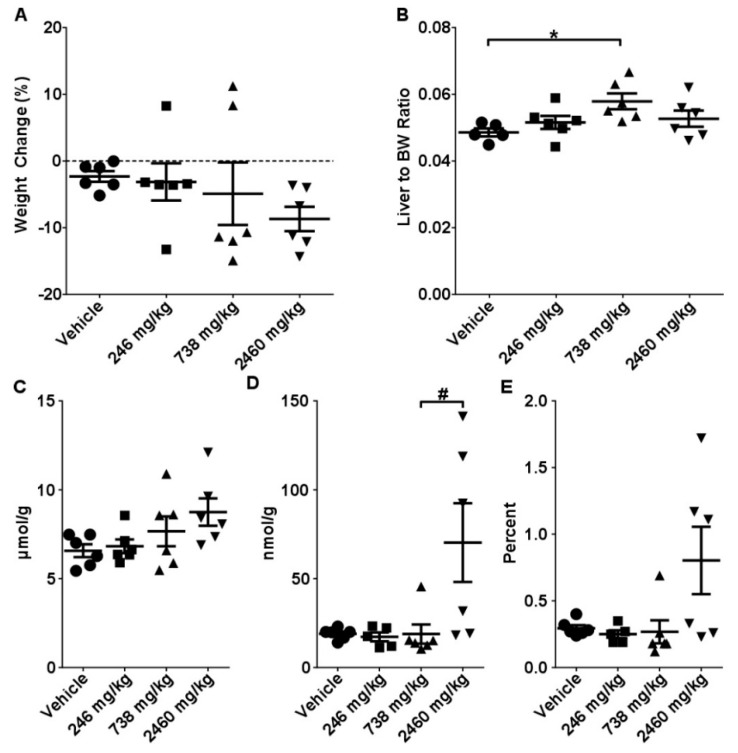
Effects of single gavage with CBD. Mice were gavaged with 246, 738, or 2460 mg/kg of CBD in sesame oil with tissues harvested at 24 h. (**A**) Body weight change, (**B**) liver to body weight ratios, intrahepatic concentrations of (**C**) total glutathione (GSH), (**D**) reduced glutathione (GSSG), and (**E**) GSH/GSSG ratio. Data are presented as mean ± SEM (*n* = 6). * indicates a significant difference as calculated with a One-Way ANOVA and Tukey’s post-hoc test, and **^#^** indicates a significant difference as calculated with a Kruskal-Wallis test with a Dunn’s post-hoc test (*p* < 0.05).

Mice at 738 mg/kg and 2460 mg/kg groups developed a sub-lethargic condition which presented as decreased appetite and slow response to exogenous stimuli at 4–5 h after CBD administration. This was still evident in mice receiving the 2460 mg/kg dose at the 24 h time-point. Administration of 2460 mg/kg CBD led to marginal body weight loss (9.5% from control) whereas the response in 738 mg/kg mice was mixed, with 4 mice exhibiting patterns of substantial weight loss (11%–15%) while two gained body weight (~10%) (Figure 1A). Further, 738 mg/kg caused significant increases in liver-to-body weight ratios (18%, *p* < 0.005), while 2460 mg/kg was characterized by an uneven distribution of these values (Figure 1B), even when using pre-gavage body weights to calculate organ to body weight ratios. No significant differences in kidney- and heart-to-body weight ratios were observed (Supplementary Figure S1A,B). No appreciable histomorphological differences were observed between control and experimental mice (Supplementary Figure S2). A small dose-dependent increase in total glutathione (GSH + GSSG) was observed in mice gavaged with CBD; however, increased levels of oxidized glutathione (GSSG) and GSSG/GSH ratio were also observed in mice gavaged with 2460 mg/kg CBD (Figure 1C–E).

Clinical biochemistry analysis revealed moderate, but statistically significant (*p* < 0.01–0.001), dose-dependent increases in both AST and ALT serum levels (Table 1, Supplementary Figure S3A,B). Administration of 2460 mg/kg CBD led to marked elevations of total bilirubin (>20-fold, *p* < 0.001) (Table 1, Supplementary Figure S3C). No significant differences were observed in ALP or GGT (Supplementary Figure S3D–E). 

**Figure 2:**
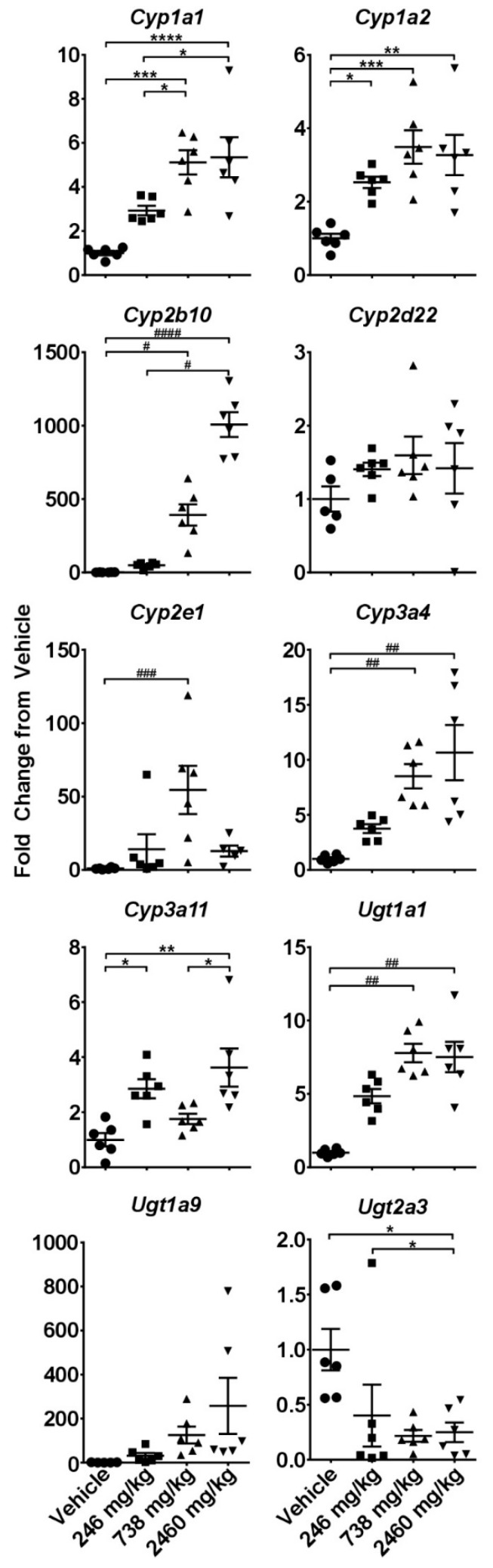
Effects of single gavage with CBD on intrahepatic expression of cytochrome P450s and UDP-glucuronosyltransferases. Livers were collected at 24 h and gene expression was measured using the quantitative real-time (qRT) PCR. * - indicate data analyzed by One-Way ANOVA with Tukey’s post-test, and **^#^** indicate non-normal data analyzed with a Kruskal-Wallis and Dunn’s post-hoc test. Data are presented as mean ± SEM fold changed from vehicle (*n* = 6), with * or ^#^ as *p* < 0.05; ** or ^##^ as *p* < 0.01; *** or ^###^ as *p* < 0.001; and **** or ^####^ as *p* < 0.0001.

Gene expression analysis of a panel of cytochromes and UDP-glucuronosyltransferases revealed significant and mostly dose-dependent increases in many isoforms (Figure 2). Of particular interest was the striking up-regulation of *Cyp2b10* (homologous to human *CYP2B6*) that exceeded 1000-fold at 2460 mg/kg CBD (*p* < 0.001). *Cyp1a2* and *Cyp2e1*, major cytochromes involved in the metabolism of ethanol and acetaminophen (APAP), were increased by 4- and 50-fold, respectively. At the same time, *Cyp2d22*, an isoform considered non-inducible, remained unaffected, lending further credence to the findings (Figure 2). 

### 2.2. Sub-Acute Toxicity Study

Given the overt toxicity observed with 2460 mg/kg, we used a starting CBD dose of 61.5 mg/kg for this phase of experiments. This dose is an MED of 5 mg/kg CBD, which it turn is analogous to initial target doses administered in clinical trials for treatment-resistant epilepsy [28]. Subsequently, doses of 184.5mg/kg and 615 mg/kg CBD were considered as 3× and 10× doses, respectively. 

Shortly after the second gavage with CBD, overt toxicity manifested as profound lethargy, loss of appetite, and body weight loss was observed in 33% of animals (2 out of 6) in the 615 mg/kg group. Two more mice developed similar symptoms after the third gavage. Thus, the remaining four animals were terminated at the end of day 3 (6 h after the third CBD dose). The remaining mice in the 61.5 mg/kg and 184.5 mg/kg cohorts were gavaged as scheduled for 10 days and exhibited no visible signs of toxicity. 

**Figure 3:**
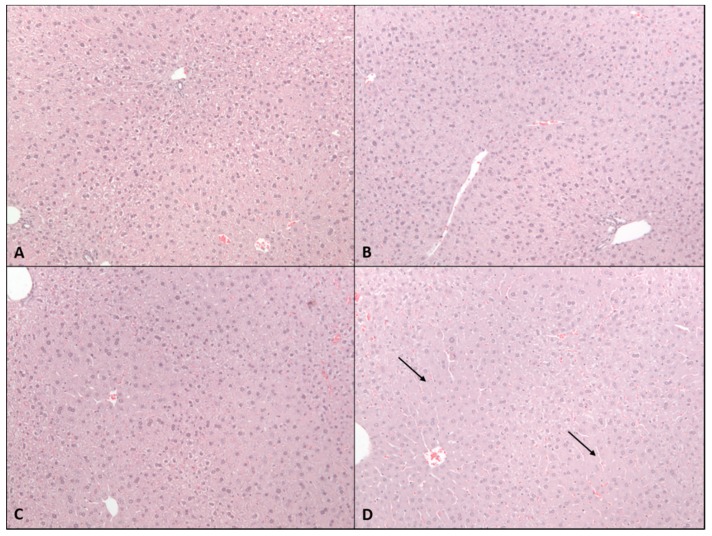
Effects of 2-week administration of CBD on liver histomorphology. H&E stained liver sections from (**A**) vehicle mice or those gavaged with (**B**) 61.5 mg/kg, (**C**) 184.5 mg/kg, or (**D**) 615 mg/kg CBD in sesame oil for 2 weeks. Note that 615 mg/kg group was terminated after 2–3 doses due to overt toxicity elicited by CBD.

Histopathological evaluation revealed pan-hepatic cytoplasmic swelling in mice gavaged with 615 mg/kg CBD (Figure 3). Foci of cytoplasmic swelling were clearly present in mice gavaged with 184.5 mg/kg, but not in the livers of mice gavaged with 61.5 mg/kg CBD. 

**Figure 4:**
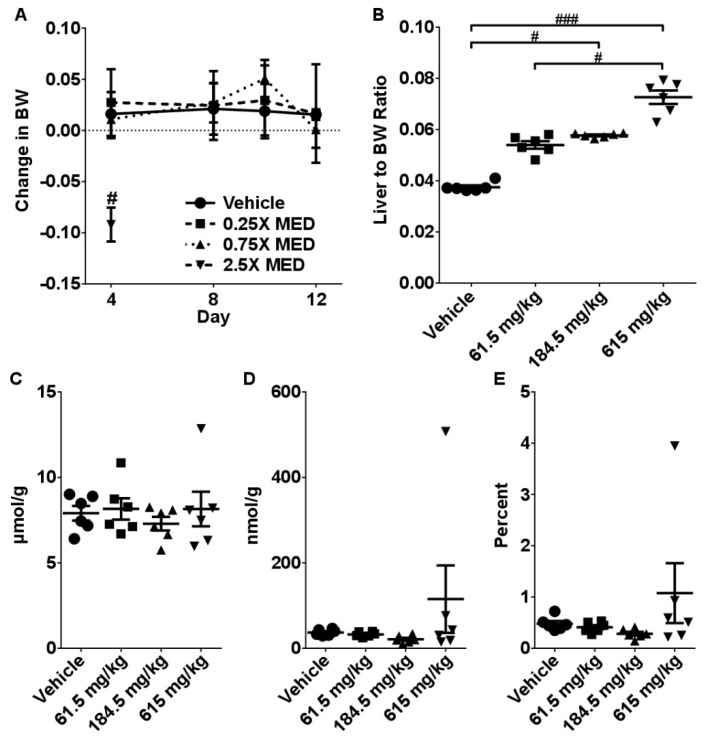
Effects of 2-week administration of CBD on: (**A**) Body weight dynamics, (**B**) Liver to body weight ratio. Intrahepatic concentrations of (**C**) total glutathione (GSH), (**D**) reduced glutathione (GSSG), and (**E**) GSH/GSSG ratio. Data are presented as mean ± SEM (*n* = 6). **^#^** indicates a significant difference as calculated with a Kruskal-Wallis test with a Dunn’s post-hoc test with ^#^ representing *p* < 0.05; and **^###^** *p* < 0.001.

Gavaging mice with 615 mg/kg CBD resulted in significant reductions in body weight (10%, *p* < 0.05) (Figure 4A). Furthermore, we observed a dose-dependent increase in liver-to- body weight ratios (5–30% range) (Figure 4B). Kidney-to-body weight ratios were significantly decreased in mice receiving 615 mg/kg CBD (Supplementary Figure S4). There were no significant differences in the total glutathione levels in any experimental groups and only modest changes were observed in GSSG and GSSG/GSH ratios (Figure 4C–E).

Analysis of clinical biochemistry parameters revealed that mice receiving 615 mg/kg CBD had significantly elevated total bilirubin, and moderately high levels of ALT and AST (Table 2, Supplementary Figure S3). However, no significant changes in any of these parameters were observed at lower CBD doses. 

**Figure 5:**
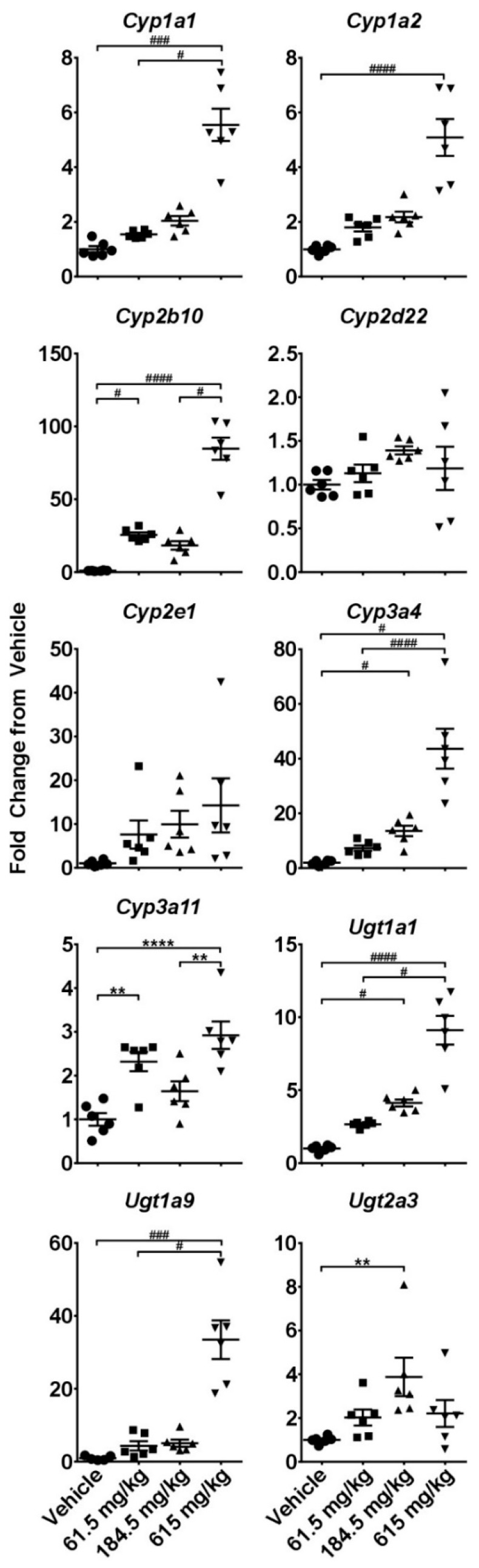
Effects of a two-week administration of CBD on intrahepatic expression of cytochrome P450s and UDP-glucuronosyltransferases. Livers were collected 6 h after the last gavage and gene expression was measured using the quantitative real-time (qRT) PCR. * - indicate data analyzed by One-Way ANOVA with Tukey’s post-test, and ^#^ indicate non-normal data analyzed with a Kruskal-Wallis and Dunn’s post-hoc test. Data are presented as mean ± SEM fold changed from vehicle (*n* = 6), with * or ^#^ as *p* < 0.05; ** or ^##^ as *p* < 0.01; *** or ^###^ as *p* < 0.001; and **** or ^####^ as *p* < 0.0001.

Gene expression analysis of a panel of cytochrome P450s (CYPs) and UDP-glucuronosyltransferases (UGTs) revealed similar patterns of response as that observed in the acute study phase. Up-regulation of CYP and UGT genes appeared dose-dependent, especially in the case of *Cyp2b10* and *Ugt1a9,* with significant changes occurring, in many instances, after the lowest CBD dose (61.5 mg/kg) (Figure 5). 

### 2.3. Hepatotoxicity Gene Expression Analysis

Given that CBD-induced hepatotoxicity was observed in both study phases, we further investigated the mechanistic underpinnings of this toxicity. For this purpose, we employed a gene expression array comprised of 84 genes recognized as biomarkers of liver toxicity. These genes have been linked to pathologies such as cholestasis, steatosis, phospholipidosis, non-genotoxic hepatocarcinogenicity, necrosis, and generalized hepatotoxicity. This approach was successful in previous studies into herb-induced liver injury [29,30,31]. 

During the acute phase, 22 genes were significantly (*p* < 0.05 ANOVA) down-regulated and 26 genes were significantly up-regulated in mice livers. The vast majority of affected genes (32) were dysregulated in a dose-dependent manner (Table 3). Furthermore, several genes, including *Fmo1*, *Lgr5*, and *Lss,* exhibited a biphasic response, where the up-regulation observed at lower doses was succeeded by down-regulation at higher dose(s). 

During the sub-acute phase, 21 genes were significantly down-regulated and 12 genes were significantly up-regulated. Unlike the acute phase, only 15 affected genes were dysregulated in a dose-dependent manner. Another 15 genes were affected only at the high CBD dose (i.e., 615 mg/kg CBD). 

Expression of a substantial number of genes (21) was affected during both study phases. The largest subset of dysregulated genes (9) was associated with general hepatotoxicity. Of these, *Aldoa*, *Gsr*, *Krt8*, *Krt18*, *Nqo1*, and *Pla2g12a* were up-regulated, whereas *Avrp1a*, *Car3*, and *Igfals* were down-regulated. 

All the gene expression data is summarized in Supplementary Table S3.

### 2.4. Dose-Response Analysis

Dose-response was also evaluated with linear and log regression models, with the best fit model meeting at least three of the following criteria: lowest AICc, lowest standard deviation of residuals, lowest absolute sum of squares, and/or highest R^2^ value (at least 0.5) (Supplementary Table S4). In the 24 h acute study, AST, *Cyp1a1*, *Cyp1a2*, *Cyp2b10*, *Cyp3a4*, *Gsr*, *Ipo4*, *Nqo1*, *Timm10b*, and *Ugt1a1* had an increasing response with CBD dose (R^2^ > 0.5) and *Abcb11*, *Car3*, *Cdc14b*, *Fabp1*, *Fads1*, *Igfals*, *L2hgdh*, *Lgr5*, *Maob*, *Mbl2*, and *Ppara* had a decreasing response with CBD dose (R^2^ > 0.5). Similar responses for some of the parameters were seen in the two-week acute study: increasing responses in *Cyp2b10*, *Ugt1a1*, and liver to body weight ratios; and decreasing responses with *Atp8b1*, *Avpr1a*, *Car3*, *Cdc14b*, *Cxcl12*, *Fabp1*, *L2hgdh*, *Lgr5*, *Ppara*, *Scd1*, and *Tagln*. The parameters in common between the two time points were *Cyp2b10*, *Ugt1a1*, *Car3*, *Fabp1*, *L2hgdh*, *Lgr5*, and *Ppara*, which, aside from *Cyp2b10* and *Ugt1a1*, were down-regulated.

## 3. Discussion

The marketing of products containing CBD, a non-psychotropic constituent of the *Cannabis sativa* plant, has grown rapidly in the last five years. It has been successfully utilized for therapy of treatment-resistant epilepsy and may have a number of other beneficial health effects. However, to our knowledge, there is a lack of comprehensive toxicological studies devoted to CBD safety that are critical for further marketing of CBD and CBD-containing products. 

In this study, we demonstrated that CBD, when delivered orally to mice in the form of a concentrated CBD-enriched Cannabis extract, has the potential to cause liver injury. In the acute toxicity study, the highest CBD dose (2460 mg/kg), exhibited clear evidence of hepatotoxicity as indicated by marked increases in serum ALT, AST, and total bilirubin as well as increased intrahepatic concentrations of oxidized glutathione. Interestingly, this dose did not result in consistent increases in liver-to-body weight ratio; however, a similar response was observed in rhesus monkeys injected with sub-lethal or lethal doses of CBD [14]. Although 2460 mg/kg (MED of 200 mg/kg CBD) is not applicable to most real-life scenarios, it does provide critical information regarding the potential consequences of CBD overdose as well as for doses needed for further sub-chronic and chronic toxicity studies. Single administration of lower doses (246 mg/kg and 738 mg/kg CBD) caused only increases in liver-to-body weight ratios among the generally liver-focused toxicological responses measured. 

The administration of CBD caused dose-dependent and sometimes dramatic induction of major cytochromes and UDP-glucuronosyltransferases (Supplemental Table S3). Induction of murine Cyp isoforms by CBD has been noted previously following sub-chronic dosing [19]. Of particular concern is the induction of *Cyp2e1* and *Cyp2b10*. The former isoform is a central participant in the biotransformation of ethanol and APAP, while the latter plays a role in the metabolism of a number of prescription medications including bupropion, clobazam, cyclophosphamide, ketamine, propofol, and several others. Furthermore, CYP2B6 and CYP3A4, the human homologues of *Cyp2b10* and *Cyp3a11* (another CYP induced in this study), are central in the metabolism (*N*-demethylation) of clobazam, an anti-seizure medication used in the treatment of epilepsy. Interestingly, recent clinical studies have noted that serum concentrations of *N*-desmethylclobazam, the active metabolite of clobazam, are markedly increased when co-administered with CBD (Epidiolex^®^) [32]. Such clinical observations appear to support the inductive effects of CBD on CYPs noted in this study. To what extent, however, the induction of murine Cyps by CBD is translatable to humans remains to be determined. Clearly, additional clinical studies investigating CBD-mediated drug interactions are needed, especially if Epidiolex^®^ is to be prescribed for other medical conditions, but more importantly as CBD gains popularity across the U.S. following its deschedulization as a result of the passage of the Agriculture Improvement Act of 2018, otherwise known as the 2018 Farm Bill [33].

The 10 day sub-acute study also revealed that CBD doses above 50 mg/kg MED, although well tolerated after single administration, were toxic when repetitively delivered. The observed general toxicity was, in part, mediated by liver injury as numerous signatures of hepatotoxicity were observed, including pan-hepatic cytoplasmic swelling, increases in liver-to-body weight ratios, and elevated ALT, AST, and total bilirubin. No measurable toxicological responses associated with liver injury were observed in mice gavaged with CBD at 184.5 mg/kg (MED of 15 mg/kg CBD) or lower, however, foci of hepatocyte cytoplasmic swelling were often detected. These findings are in line with observations from recent clinical trials in which 5–20% of patients exhibited increases in liver enzymes during chronic CBD administration at doses of 20 mg/kg [1,2,24]. Taken together, this evidence suggests that, despite some inter-species differences in CBD disposition, the mouse is a reliable model for assessing the safety of this popular cannabinoid. 

Another important finding of this study was the wide palette of molecular responses elicited by CBD, particularly the dysregulation of more than 50 genes involved in hepatotoxicity. To our knowledge, the magnitude of such a response has not been observed in previous studies utilizing similar gene expression arrays aimed at examining the hepatotoxicity of bromobenzene, carbon tetrachloride, dimethyl nitrosamine, or OxyELITE-Pro, a botanical dietary supplement linked to severe liver injury in humans [29,30,34]. The involvement of numerous enzymatic pathways in response to CBD exposure suggests that liver injury associated with this cannabinoid occurs via various mechanisms. Of particular concern was the up-regulation of genes associated with oxidative stress, in particular *Hmox1*, *Nqo1*, and *Txnrd1*. These findings, coupled with increased levels of oxidized glutathione, infer a strong pro-oxidant trait to CBD, thereby bringing into question its claimed ‘antioxidant’ properties.

Importantly, a number of genes were differentially regulated at low and high doses of CBD, resulting in a biphasic or hormetic response. For instance, the expression of lanosterol synthase (*Lss)*, a gene responsible for the biosynthesis of cholesterol, steroid hormones, and vitamin D, was up-regulated after 246 and 738 mg/kg CBD, but substantially down-regulated with a CBD dose of 2460 mg/kg. Interestingly, previous studies have also described a biphasic response to CBD, where low doses were stimulatory, while higher doses were inhibitory [35]. More strikingly, cyclin dependent kinase inhibitor 1A (*Cdkn1a*) was down-regulated after exposure to non-toxic doses of CBD, but significantly up-regulated at doses associated with either overt toxicity (2460 mg/kg CBD) or mortality (615 mg/kg CBD) in acute and sub-acute studies, respectively. Previous studies have demonstrated that down-regulation of *Cdkn1a*, also known as *p21*, stimulates liver regeneration, while overexpression inhibits this process [36,37,38]. Furthermore, a recent report of *Cdkn1a* up-regulation resulting from acute cholestatic injury was proposed as a biomarker for impaired liver regeneration [39]. Elevated total bilirubin in conjunction with up-regulation of *Cdkn1a* and a number of other gene-markers of cholestatic liver injury (i.e., *Abcb1a*, *Abcc2*, *Abcc3*, *Atp8b1*, and *Rdx*) and down-regulation of fatty acid metabolism-related genes (*Car3*, *Fabp1*, and *Ppara*) observed in our study, suggest that CBD-induced liver injury may be cholestatic, though ALP and GGT were not elevated. Future studies are needed to confirm this hypothesis. 

In conclusion, the results of these studies demonstrate that, despite the beneficial effects of CBD in the treatment of certain therapy-resistant seizures, it poses a risk for liver injury. Furthermore, the probability of CBD-drug interactions appears quite high. Therefore, additional studies are needed to examine the toxicity of chronic low-dose CBD exposure as well as explore CBD’s potential to interact with other medications. Such studies will provide important information regarding the range of CBD doses that can be deemed safe for the purpose of regulatory decision-making. 

## 4. Materials and Methods 

### 4.1. CBD Extract Characterization, Dosing Solution, and Dose Calculations

CBD extract was prepared following GMP procedures from the leaves and flowering tops by the extraction of CBD rich cannabis plant material (5.61% of CBD and 0.2% THC) using hexane as the extraction solvent. The extract was then evaporated to dryness followed by raising the temperature to 80 °C to effect complete decarboxylation of the extract. The final extract was analyzed using GC/MS for its cannabinoid content, solvent residue, heavy metals, bacterial and fungal counts and aflatoxin content following USP procedures. The results showed the following: cannabidiol content 57.9%; other cannabinoids: cannabichromene 2.03%, Δ^9^-tetrahydrocannabinol 1.69%, cannabigerol 1.07%, Δ^8^-tetrahydrocannabinol <0.01%; tetrahydrocannabivarin <0.01%. Residual solvent <0.5%; loss on drying 0.32%; heavy metals: lead, mercury, cadmium, and arsenic were not detected; aflatoxins: AFB_1_, AFB_2_, AGF_1_, AFG_2_ were not detected. 

Doses of the CBD extract were calculated based on the CBD content listed above to deliver the required dose of CBD. For simplicity, the ‘CBD-rich cannabis extract’ will be referred to as ‘CBD’ throughout this manuscript. The extract was diluted in sesame oil to prepare the gavage solution. Allometric scaling for CBD mouse equivalent doses (MED) was determined per the recommendation of Wojcikowski and Gobe which, in turn, is based upon the FDA Industry Guidance for Estimating the Maximum Safe Starting Dose in Initial Clinical Trials for Therapeutics in Adult Volunteers [40]. The scaling factor of 12.3, commonly used for mice weighing between 11–34 g, was used to calculate the MED for CBD. The MED was based on the maximum recommended human maintenance dose of CBD (Epidiolex^®^), which is 20 mg/kg. For the 1× dose, the quantity of CBD administered was 20 mg/kg × 0.025 kg (average mouse weight in our study) × 12.3 (scaling factor for mice) = 6.15 mg total CBD delivered in 300 µL of gavage solution or 246 mg/kg. Consequently, 3× dose = 18.45 mg total CBD in 300 µL gavage solution or 738 mg/kg), and 10× dose = 61.5 mg total CBD in 300 µL gavage solution or 2460 mg/kg). In the sub-acute study, the dose of 61.5 mg/kg (MED of 5 mg/kg CBD) was considered as 1× dose. Consequently, the doses of 184.5 mg/kg (MED of 15 mg/kg CBD) and 615 mg/kg (MED of 50 mg/kg CBD) were considered as 3× and 10×, respectively. Control mice received 300 µL of sesame oil. 

### 4.2. Animals

Male B6C3F_1_/J mice, 8 weeks of age (standard age of mice used in safety assessment studies), were purchased from Jackson Laboratories (Bar Harbor, ME, USA) and were housed at the UAMS Division of Laboratory Animal Medicine (DLAM) facility. B6C3F_1_/J mice are characterized by an average sensitivity to hepatotoxicants and are widely used by both the FDA and pharmaceutical industry to investigate the potential for xenobiotics to produce hepatotoxicity. Animals were given one week to acclimate before the initiation of studies. Animal experiments were conducted in two stages. In the first stage (acute toxicity), mice were gavaged with a single dose of either 246, 738, or 2460 mg/kg of CBD (MED of 20, 60, and 200 mg/kg, respectively) and 24 h later animals were euthanized and tissues/organs were harvested. During the second stage (sub-acute toxicity), mice were gavaged with CBD extract for ten days (Mon-Fri) with (MED of 61.5, 184.5, and 615 mg/kg, respectively) for reasons explained later in the Results. Mice were terminated six hours after the last gavage. 

To avoid any potential fasting-exacerbated toxicity, food and water were provided *ad libitum*. Each animal was individually identified with an ear tag. Animal body weights were measured and recorded twice a week. All procedures were approved by the UAMS Institutional Animal Care and Use Committee (protocol number: AUP # 3701), and all personnel followed the appropriate safety precautions.

### 4.3. Blood Sampling and Clinical Biochemistry

To measure the effects of CBD extract on a panel of liver enzymes characteristic for drug-induced liver injury, blood was collected at the end of each experimental stage described above. Blood was collected from the retroorbital plexus with a heparinized micro-haematocrit capillary tube (Fisher Scientific, Pittsburg, PA, USA) and placed into a 1.1 mL Z-gel microtube (Sarstedt, Newton, NC, USA). Tubes were kept on ice and centrifuged at 10,000 rpm for 20 min; serum samples were then immediately aliquoted and delivered to the Veterinary Diagnostic Laboratory at the Arkansas Livestock and Poultry Commission (Little Rock, AR, USA) on dry ice where the samples were processed the same day. 

### 4.4. Histopathological Assessment

Livers were excised and a 1 mm section was obtained from the left lateral lobe and another from the right medial lobe. Sections were fixed in 4% formalin for 24 h, then briefly rinsed in PBS and stored in 70% ethanol for 24 h. Livers were then processed at the UAMS Pathology Core Facility, stained with hematoxylin eosin, and evaluated by a board-certified pathologist in a blinded fashion. 

For histologic assessment purposes, each liver was represented by two sections obtained from different locations within the liver. Each section was initially evaluated at magnifications of ×40 and ×100. Sections were further evaluated at ×200 and ×400 to check for the presence of mitotic figures, necrotic foci, and apoptotic bodies.

### 4.5. Glutathione Analysis

Glutathione was measured using a modified Tietze assay [41]. Briefly, liver tissue was homogenized in 3% sulfosalicylic acid. One aliquot was diluted in N-ethylmaleimide (NEM) to mask reduced glutathione (GSH) and facilitate measurement of oxidized glutathione (GSSG), while another was diluted in 0.1 M HCl for measurement of total (GSH + GSSG) glutathione. After removal of NEM by solid phase extraction with a C18 column, glutathione was measured in both aliquots using a colorimetric glutathione reductase cycling detection method [41].

### 4.6. Analysis of mRNA Levels of Major Cytochromes and Transporter Genes 

Total RNA was extracted from flash frozen liver tissue using the RNeasy Mini Kit (QIAGEN, Germantown, MD, USA). Following purification, 1000 ng were reverse transcribed with the High Capacity cDNA Reverse Transcription Kit (Thermo Fisher, Waltham, MA, USA). Primers were added at a final concentration of 5 µM (Supplementary Table S1). Gene expression values were normalized to the internal control gene *Hprt* and expressed as fold change according to the ΔΔ*C*t method.

### 4.7. Hepatotoxicity Gene Expression Array

Total RNA was extracted as described above. The cDNA was diluted to 5 ng/µL and 105 µL was mixed with an equal volume of 2× TaqMan^®^ Fast Advanced Master Mix. For real-time PCR, 100 µL of the mix was applied to each of two channels on a TaqMan Low Density Hepatotoxicity Array (TLDA) (Supplementary Table S2) (Thermo Fisher, Waltham, MA, USA). Four biological samples were loaded onto each array with six replicates analyzed per group. Analysis was performed using the ExpressionSuite Software v1.1 (Thermo Fisher, Waltham, MA, USA).

### 4.8. Statistical Analysis

All statistical analyses were performed with Graphpad Prism 6 software (Graphpad Software, San Diego, CA, USA). Treatment groups were compared with their respective untreated group using ANOVA followed by Tukey’s multiple comparison test. In cases where the data was not normally distributed as indicated by a positive Brown-Forsythe test, a Kruskal-Wallis with Dunn’s multiple comparison test was used instead. Comparisons were considered significant at *p* < 0.05. To evaluate trends in dose-dependent responses, all parameters were analyzed with regression analyses within Prism using log(dose) as the independent variable. We used three different models to determine best fit: linear regression with y-intercept constrained to 0, linear regression with unconstrained y-intercept, and log(agonist) vs response (three parameters). Regression models were excluded if the model was ambiguous, interrupted, or not converged or if the 95% confidence intervals were calculated to be ‘very wide’. Models for each response parameter were compared using the goodness of fit parameters R^2^ (higher being better), standard deviation of residuals (lower being better; noted as Sy.x), AICc (lower being better), and the absolution sum of squares (lower being better), with the model having best goodness of fit parameters being considered the better model.

## Figures and Tables

**Table 1:** Clinical chemistry parameters 24 h after dosing with CBD oil. Cells in bold italics are significantly different from vehicle (One-Way ANOVA, indicated by *, or Kruskal-Wallis with appropriate post-hoc test). Data presented as mean ± SEM (*n* = 6/group).

	Vehicle	246 mg/kg	738 mg/kg	2460 mg/kg
ALT	27.5 ± 2.7	31.7 ± 2.6	38.7 ± 4.3 *	57.0 ± 8.0 *
AST	51.0 ± 2.0	58.7 ± 1.8	80.3 ± 3.7 *	120.8 ± 14.3 *
ALP	110.8 ± 2.9	106.0 ± 4.5	90.3 ± 8.8	98.8 ± 4.2
GGT	4.7 ± 0.2	4.3 ± 0.5	5.5 ± 0.3	4.2 ± 0.8
Total Bilirubin	0.1 ± 0.0	0.1 ± 0.0	0.13 ± 0.0	***2.1 ± 0.1***

**Table 2:** Clinical chemistry parameters after dosing with CBD oil for two weeks. Cells in bold italics are significantly different from vehicle (One-Way ANOVA, indicated by *, or Kruskal-Wallis with appropriate post-hoc test). Data presented as mean ± SEM (*n* = 6/group).

	Vehicle	61.5 mg/kg	184.5 mg/kg	615 mg/kg
ALT	40.0 ± 3.6	41.3 ± 14.8	32.3 ± 4.3	115.4 ± 51.2
AST	66.7 ± 6.7	74.7 ± 6.7	68.2 ± 3.9	157.0 ± 48.9
ALP	112.2 ± 3.5	112.7 ± 4.6	104.0 ± 2.0	113.2 ± 12.2
GGT	5.2 ± 0.3	4.0 ± 0.4	4.7 ± 0.2	5.0 ± 0.3
Total Bilirubin	0.1 ± 0.0	0.1 ± 0.02	0.23 ± 0.02	1.5 ± 0.7 *

**Table 3:** Gene-markers of hepatotoxicity affected by CBD administration. Genes that are significantly up- or down-regulated sorted by greatest fold changed at the highest dose (2460 mg/kg or MED of 200 mg/kg). Cells in bold italics indicate those significantly different from vehicle (One-Way ANOVA, indicated by *, or Kruskal-Wallis test with appropriate post-hoc comparison). Genes that are commonly and significantly dysregulated in both the acute and sub-acute studies are highlighted in yellow. Data presented as mean ± SEM (*n* = 6/group) fold change from vehicle.

**Up-Regulated**						
	**Single Dose**			**2 Week Dosing**		
**Gene**	**246 mg/kg**	**738 mg/kg**	**2460 mg/kg**	**61.5 mg/kg**	**184.5 mg/kg**	**615 mg/kg**
*Krt8*	32.6 ± 18.5	***32.2 ± 0.4***	***46.9 ± 25.1***	0.7 ± 0.1	0.8 ± 0.2	8.7 ± 4.2 *
*Map3k6*	4.9 ± 1.9	1.6 ± 0.2	***38.0 ± 17.3***	1.7 ± 0.9	0.8 ± 0.1	***8.6 ± 2.9***
*Cdkn1a*	***0.3 ± 0.1***	0.5 ± 0.2	***22.7 ± 6.9***	***0.3 ± 0.1***	0.4 ± 0.1	**9.3 ± 3.7**
*Hmox1*	0.9 ± 0.1	2.8 ± 0.6	19.0 ± 8.8 *	1.5 ± 0.4	1.5 ± 0.3	2.4 ± 1.2
*Nqo1*	3.6 ± 0.8	4.92 ± 1.0 *	9.7 ± 1.7 *	1.0 ± 0.1	1.1 ± 0.1	6.2 ± 1.2 *
*Ugt1a1*	4.8 ± 0.5	***7.8 ± 0.6***	***7.5 ± 1.0***	2.7 ± 0.1	***4.1 ± 0.2***	***9.1 ± 0.9***
*Rplp2*	1.0 ± 0.1	1.5 ± 0.2	5.7 ± 0.8 *	0.5 ± 0.1	0.5 ± 0.1	0.7 ± 0.2
*Abcb1a*	1.5 ± 0.4	1.0 ± 0.2	***5.6 ± 0.8***	2.1 ± 0.5	2.1 ± 0.2	6.4 ± 1.8 *
*Slc39a6*	2.0 ± 0.3	1.4 ± 0.1	***5.5 ± 1.3***	1.1 ± 0.2	0.9 ± 0.1	2.9 ± 1.1
*Aldoa*	2.8 ± 0.9	2.1 ± 0.2	***5.4 ± 1.4***	1.3 ± 0.1	1.1 ± 0.1	2.8 ± 0.5 *
*Pla2g12a*	1.6 ± 0.3	2.0 ± 0.3	***5.3 ± 0.6***	1.7 ± 0.3	1.4 ± 0.1	***3.5 ± 0.3***
*Ubc*	1.3 ± 0.1	1.1 ± 0.2	4.8 ± 0.6 *	0.6 ± 0.1	1.1 ± 0.1	1.6 ± 0.3
*Abcc3*	2.6 ± 0.6	2.4 ± 0.4	***4.5 ± 1.2***	1.2 ± 0.2	1.1 ± 0.1	2.2 ± 0.3 *
*Abcc2*	1.4 ± 0.2	1.6 ± 0.3	3.9 ± 0.3 *	1.2 ± 0.1	0.9 ± 0.0	3.4 ± 1.3
*Gsr*	1.9 ± 0.5	2.7 ± 0.3 *	3.9 ± 0.4 *	0.8 ± 0.1	0.9 ± 0.1	2.9 ± 0.5 *
*Icam1*	1.9 ± 0.4	1.2 ± 0.2	***3.8 ± 0.9***	0.8 ± 0.1	0.7 ± 0.1	1.9 ± 0.6
*Cyp3a11*	2.9 ± 0.3 *	1.6 ± 0.2	3.6 ± 0.7 *	2.3 ± 0.2 *	1.6 ± 0.2	2.9 ± 0.3 *
*Txnrd1*	1.3 ± 0.2	1.6 ± 0.3	***3.5 ± 0.4***	1.4 ± 0.3	1.0 ± 0.2	2.6 ± 1.0
*Lss*	***3.2 ± 0.5***	***2.2 ± 0.1***	***0.3 ± 0.0***	0.5 ± 0.2	0.4 ± 0.2 *	0.9 ± 0.7
*Pgk1*	1.7 ± 0.4	1.3 ± 0.1	***3.1 ± 0.9***	0.9 ± 0.0	1.0 ± 0.1	1.6 ± 0.3
*Ddx39*	1.1 ± 0.1	2.2 ± 0.6 *	2.6 ± 0.3 *	0.9 ± 0.1	0.6 ± 0.1	3.1 ± 0.7
*Psme3*	1.2 ± 0.2	1.0 ± 0.1	***2.6 ± 0.5***	1.0 ± 0.1	0.8 ± 0.1	1.5 ± 0.3
*Ipo4*	1.1 ± 0.1	1.9 ± 0.3 *	2.5 ± 0.2 *	0.8 ± 0.1	0.9 ± 0.1	1.7 ± 0.4
*Osmr*	1.4 ± 0.2	1.0 ± 0.2	2.4 ± 0.3 *	0.7 ± 0.1	0.4 ± 0.1	1.5 ± 0.6
*Krt18*	1.5 ± 0.2	2.0 ± 0.2 *	2.2 ± 0.4 *	0.7 ± 0.1	0.7 ± 0.1	3.2 ± 0.9
*Timm10b*	1.3 ± 0.1	1.7 ± 0.2 *	1.9 ± 0.3 *	1.0 ± 0.1	1.1 ± 0.2	1.7± 0.4
*Tfrc*	2.0 ± 0.1	***3.3 ± 0.6***	1.8 ± 0.1	1.3 ± 0.1	0.7 ± 0.1	2.6 ± 0.3 *
*Mrps18b*	0.8 ± 0.0	0.9 ± 0.1	1.7 ± 0.2 *	1.0 ± 0.1	0.8 ± 0.1	1.6 ± 0.3
**Down-Regulated**						
	**Single Dose**			**2 Week Dosing**		
**Gene**	**20 mg/kg**	**60 mg/kg**	**200 mg/kg**	**5 mg/kg**	**15 mg/kg**	**50 mg/kg**
*Igfals*	1.1 ± 0.1	0.5 ± 0.1 *	0.02 ± 0.0 *	0.8 ± 0.1	0.9 ± 0.2	0.2 ± 0.1 *
*Lgr5*	1.2 ± 0.1	0.5 ± 0.1 *	0.04 ± 0.0 *	0.4 ± 0.0 *	0.3 ± 0.1 *	0.2 ± 0.0 *
*Car3*	0.8 ± 0.1	0.2 ± 0.0 *	0.1 ± 0.1 *	0.5 ± 0.1 *	0.5 ± 0.0 *	0.1 ± 0.0 *
*Atp8b1*	1.4 ± 0.3	0.5 ± 0.1	***0.2 ± 0.0***	0.6 ± 0.1	0.5 ± 0.1	***0.4 ± 0.1***
*Ppara*	0.7 ± 0.1*	0.6 ± 0.0 *	0.2 ± 0.0 *	0.5 ± 0.1 *	0.5 ± 0.1 *	0.3 ± 0.1 *
*Avpr1a*	1.1 ± 0.2	0.8 ± 0.1	0.2 ± 0.0 *	0.5 ± 0.1 *	0.3 ± 0.1 *	0.3 ± 0.1 *
*Abcb11*	1.1 ± 0.2	0.5 ± 0.0 *	0.2 ± 0.0 *	0.6 ± 0.1	0.7 ± 0.1	0.3 ± 0.1 *
*Mcm10*	1.9 ± 0.5	1.4 ± 0.1	0.2 ± 0.0	0.9 ± 0.2	0.6 ± 0.1	0.2 ± 0.0 *
*Fabp1*	0.6 ± 0.0 *	0.4 ± 0.0 *	0.2 ± 0.1 *	0.8 ± 0.1	0.7 ± 0.0 *	0.1 ± 0.0 *
*Fads1*	0.7 ± 0.1	0.6 ± 0.0	***0.2 ± 0.0***	0.9 ± 0.1	0.8 ± 0.1	0.6 ± 0.2
*Cdc14b*	0.9 ± 0.1	0.7 ± 0.1	***0.3 ± 0.0***	0.6 ± 0.1 *	0.7 ± 0.1	0.4 ± 0.1 *
*Mbl2*	1.3 ± 0.1	0.7 ± 0.0	0.3 ± 0.0 *	0.9 ± 0.1	0.9 ± 0.1	0.6 ± 0.1 *
*Asah1*	1.1 ± 0.1	0.9 ± 0.1	0.4 ± 0.1 *	0.8 ± 0.1	0.7 ± 0.1	0.6 ± 0.1
*Lpl*	0.7 ± 0.0	0.4 ± 0.1	0.5 ± 0.1	0.4 ± 0.1	0.5 ± 0.1	***0.2 ± 0.1***
*Emc9*	1.3 ± 0.2	0.7 ± 0.0	***0.5 ± 0.0***	0.9 ± 0.1	0.9 ± 0.1	0.6 ± 0.1
*Rhbg*	0.9 ± 0.1	0.5 ± 0.0 *	0.5 ± 0.1 *	0.7 ± 0.1	1.0 ± 0.1	0.6 ± 0.2
*L2hgdh*	0.9 ± 0.1	0.8 ± 0.0	0.5 ± 0.1 *	0.7 ± 0.1	0.7 ± 0.1	0.4 ± 0.1 *
*Cxcl12*	1.2 ± 0.2	0.8 ± 0.1	0.5 ± 0.1	0.8 ± 0.0	0.7 ± 0.0 *	0.4 ± 0.1 *
*Maob*	1.0 ± 0.1	0.8 ± 0.1	0.6 ± 0.0 *	0.8 ± 0.1	0.8 ± 0.1	0.6 ± 0.1 *
*Rdx*	1.1 ± 0.1	0.8 ± 0.1	0.6 ± 0.0 *	0.7 ± 0.1	0.8 ± 0.1	0.7 ± 0.1
*B2m*	1.0 ± 0.1	0.6 ± 0.0 *	0.7 ± 0.1	0.9 ± 0.1	0.8 ± 0.1	0.6 ± 0.1 *
*Cryl1*	1.0 ± 0.2	0.4 ± 0.0	***0.7 ± 0.1***	0.8 ± 0.1	0.8 ± 0.1	0.6 ± 0.1
*Ipo8*	1.4 ± 0.1	0.8 ± 0.1 *	0.9 ± 0.1	0.9 ± 0.1	0.7 ± 0.1	0.5 ± 0.2 *
*Srebf1*	1.2 ± 0.1	0.7 ± 0.1	0.9 ± 0.1	0.8 ± 0.1	0.7 ± 0.1	0.7 ± 0.1 *
*Scd1*	0.6 ± 0.1	0.3 ± 0.0 *	0.9 ± 0.3	0.5 ± 0.1	0.6 ± 0.0	***0.1 ± 0.0***
*Dnajb11*	1.3 ± 0.2	1.1 ± 0.1	1.1 ± 0.2	0.7 ± 0.1	0.4 ± 0.1 *	0.9 ± 0.1
*Tagln*	1.0 ± 0.1	0.8 ± 0.2	1.2 ± 0.7	0.6 ± 0.1	0.5 ± 0.1 *	0.3 ± 0.1 *
*Abcb4*	1.1 ± 0.3	0.6 ± 0.1	1.4 ± 0.4	0.8 ± 0.1	0.6 ± 0.1	0.4 ± 0.1 *
*Fasn*	2.2 ± 0.3	1.4 ± 0.1	1.5 ± 0.5	0.5 ± 0.0	0.4 ± 0.1 *	0.5 ± 0.2

## Supplementary Materials

The following are available online, Figure S1: Kidney- and Heart-to Body-Weight Ratio in Mice Gavaged with Single Dose of CBD, Figure S2: Effects of Single Dose of CBD on Liver Histomorphology, Figure S3: Kidney- and Heart-to-Body-Weight Ratio in Mice Gavaged with CBD for 10 Days, Figure S4: Kidney- (A) and heart- (B) to-body-weight ratio in mice gavaged with CBD for 10 days, Table S1: Forward and Reverse Primer Sequences for Cyp P450s and UGTs, Table S2: Custom Taqman Low Density Hepatotoxicity Array Gene Targets, Table S3: Commonly dysregulated genes sorted by function, including CYPs, Ugts, and hepatotoxicity markers, Table S4: Dose-response analysis with linear and log regression models.
