# Supplementary material for: Hepatotoxicity of a Cannabidiol-Rich Cannabis Extract in the Mouse Model
Source: Molecules. 2019 Apr 30;24(9):1694. doi: 10.3390/molecules24091694 (PMC6539990; doi:10.3390/molecules24091694)
Supplement: Supplementary file 1 [file molecules-24-01694-s001.zip › Supplementary Figure and Table Legeneds - revised.docx]

**Supplementary Figure 1. Kidney- (A) and heart- (B) to-body-weight ratio in mice gavaged with single dose of CBD.** Data are presented as mean ± SEM (n = 6).

**Supplementary Figure 2. Effects of single dose of CBD on liver histomorphology.** H&E stained liver sections from A) vehicle mice or those gavaged with B) 246 mg/kg, C) 738 mg/kg, or D) 2460 mg/kg of CBD in sesame oil.

**Supplementary Figure 3.** Clinical chemistry parameters for acute and sub-acute dosing of CBD. Data are presented as mean ± SEM (n = 6). ***** indicates a significant difference as calculated with a One-Way ANOVA and Tukey post-hoc test (*p* < .05).

**Supplementary Figure 4. Kidney- (A) and heart- (B) to-body-weight ratio in mice gavaged with CBD for 10 days.** Data are presented as mean ± SEM (n = 6). ***** indicates a significant difference as calculated with a One-Way ANOVA and Tukey post-hoc test, and **^#^** indicates a significant difference as calculated with a Kruskall-Wallis test with a Dunn’s post-hoc test (*p* < .05).

**Supplementary Table 1.** Forward and reverse primer sequences for cytochrome P450s and UDP-gluconosyltransferases.

**Supplementary Table 2.** Taqman Custom Array Targets.

**Supplementary Table 3.** Commonly dysregulated genes sorted by function, including CYPs, Ugts, and hepatotoxicity markers.

**Supplementary Table 4.** Dose-response analysis with linear and log regression models.
