# Supplementary material for: Hepatotoxicity of a Cannabidiol-Rich Cannabis Extract in the Mouse Model
Source: Molecules. 2019 Apr 30;24(9):1694. doi: 10.3390/molecules24091694 (PMC6539990; doi:10.3390/molecules24091694)
Supplement: Supplementary file 1 [file molecules-24-01694-s001.zip › Table S1.docx]

|  | Forward | Reverse |
| --- | --- | --- |
| *Cyp1a1* | tgcccttcattggtcacatg | cacgtccccatactgctgact |
| *Cyp1a2* | gacatggcctaacgtgcag | ggtcagaaagccgtggttg |
| *Cyp2b10* | aaggagaagtccaaccagca | ctctgcaacatgggggtact |
| *Cyp2d22* | cagtggttgtactaaatgggct | gctaggactataccttgagagcg |
| *Cyp2e1* | tccctaagtatcctccgtga | gtaatcgaagcgtttgttga |
| *Cyp3a4* | Aaagccgcctcgattctaagc | actacatcccgtggtacaacc |
| *Cyp3a11* | acaaacaagcagggatggac | ggtagaggagcaccaagctg |
| *Ugt1a1* | CACCTGAAGCCTCAATACCAT | CAGTCCGTCCAAGTTCCACC |
| *Ugt1a6* | ATACCATGGGAGCCAGAGTG | ACCAGAACTGTGAGGGTTGG |
| *Ugt1a9* | CTGGTTCAGCCAGAGGTTTC | TTGGCGACAATTAATCCACA |
| *Ugt2a3* | CCCAGAAGGTTTTGTGGAGA | CCACCATGTGTGATGAAAGC |
| Table S1. Forward and reverse primer sequences for cytochrome P450s and UDP-gluconosyltransferases. | | |
