# Supplementary figures and images for: Hepatotoxicity of a Cannabidiol-Rich Cannabis Extract in the Mouse Model
Source: Molecules. 2019 Apr 30;24(9):1694. doi: 10.3390/molecules24091694 (PMC6539990; doi:10.3390/molecules24091694)

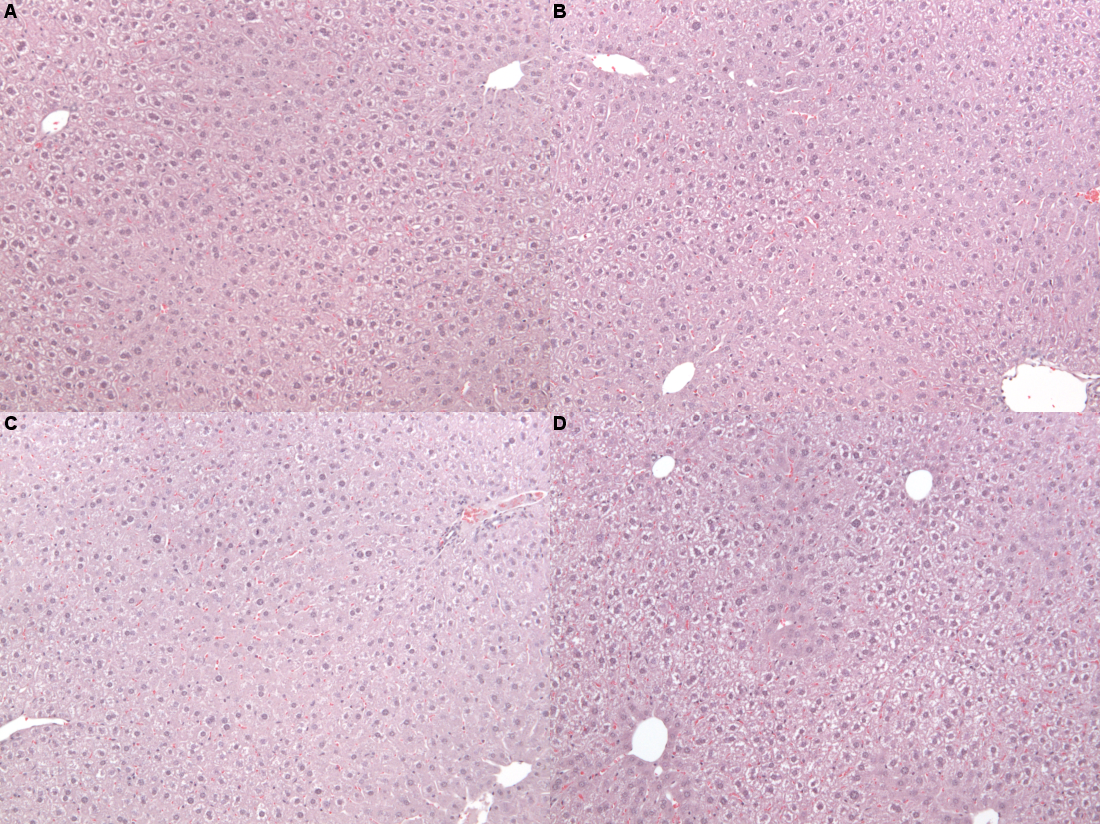

Supplement: Supplementary file 1 [file molecules-24-01694-s001.zip › FigS2.tif.tiff]
